# Clonazepam for pain due to muscle spasm in a patient with vertebral compression fractures caused by multiple myeloma: a case report

**DOI:** 10.1186/s40981-021-00477-1

**Published:** 2021-10-09

**Authors:** Kazuki Akita, Yasutomo Kumakura, Emi Nakajima, Hiroki Ishiguro, Tetsuya Iijima

**Affiliations:** 1 Department of Anesthesiology, University of Yamanashi, 1110 Shimokato, Chuo City, Yamanashi Japan; 2 Center of Medical team, University of Yamanashi Hospital, 1110 Shimokato, Chuo City, Yamanashi Japan; 3 Department of Neuropsychiatry and Clinical Ethics, University of Yamanashi, 1110 Shimokato, Chuo City, Yamanashi Japan

**Keywords:** Clonazepam, Muscle spasm, Facet joint pain, Multiple myeloma

## Abstract

**Background:**

Vertebral compression fractures can cause severe back pain. Although many types of analgesics and interventional treatments are available, they are sometimes ineffective in mitigating the pain. We encountered a case where clonazepam was effective for the management of severe low back pain caused by lumbar vertebral compression fractures.

**Case presentation:**

A 44-year-old male was diagnosed with multiple myeloma and had vertebral compression fractures of the first and second lumbar vertebrae. He had been suffering from severe low back pain on movement with muscle spasm and pain-associated anxiety. We considered this breakthrough low back pain to be caused by facet joint pain; thus, we prescribed clonazepam as a muscle relaxant and anxiolytic. Following this treatment, the intractable breakthrough pain was dramatically relieved.

**Conclusion:**

Clonazepam, which has both muscle relaxant and anxiolytic effects, might be helpful in mitigating pain, associated anxiety, and muscle spasms due to vertebral compression fractures.

## Background

Vertebral compression fractures (VCFs) are often caused by trauma or weakening of the vertebrae. They are sometimes accompanied by bone metastases in patients with neoplasms. VCFs cause severe low back pain in some cases, which lead to a decline in activities of daily living. Several types of analgesics and interventional treatments are used for severe low back pain associated with VCFs; however, it is difficult to provide adequate pain relief to these patients.

Clonazepam, a benzodiazepine, is used for management of seizures, panic disorder, and akathisia. On the other hand, it is sometimes used as an adjuvant analgesic [1]. However, it is unclear whether the analgesic effects of clonazepam are limited to specific types of pain.

Here, we report a case of effective use of clonazepam for the management of refractory low back pain in a patient with VCFs due to multiple myeloma (MM).

## Case presentation

A 44-year-old male, with moderate low back pain at rest and severe low back pain on movement, was admitted to our hospital. He had been experiencing the pain for 2 weeks before the consultation. Although he was prescribed a non-steroidal anti-inflammatory drug, the analgesic effect was poor. The medical team diagnosed the patient with MM. He had pathological compression fractures on the first and second lumbar vertebrae; therefore, we attributed his low back pain to the VCFs.

The region of pain was on the right side of the fractured vertebrae, and the pain resembled muscle cramping and spasm on movement. On the numerical rating scale (NRS) ranging from 0 to 10, the patient rated the pain as 8 at rest and 10 on movement. This severe, breakthrough low back pain induced fear and anxiety, preventing the patient from sitting upright. Regarding neurological symptoms, although there was a slight numbness in the right lower limb, no obvious decrease in sensation was observed. No motor and bladder dysfunctions were observed in this patient. Blood and other laboratory tests revealed renal dysfunction, anemia, and hypercalcemia.

Computed tomography (CT) and magnetic resonance imaging (MRI) revealed change in the first and second lumbar vertebrae, resembling fish-vertebrae; however, no spinal root compression or collapse of the facet joint was observed. Bortezomib, lenalidomide, and dexamethasone therapy was initiated 7 days after consultation.

We initially prescribed acetaminophen 3000 mg/day; however, this was ineffective. Next, we administered continuous intravenous infusion of fentanyl 500 μg/day, ketamine 40 mg/day, and lidocaine 400 mg/day using a patient-controlled analgesia pump (i-Fusor Plus™; JMS Co., Ltd., Tokyo, Japan). His pain at rest was mitigated and rated 3 on the NRS. Nevertheless, the pain on movement remained unchanged even after the fentanyl dose was increased to 700 μg/day, including bolus administration. This severe breakthrough pain escalated the patient’s stress, inducing presyncope. The patient and his family refused treatment for cancer and strongly urged for pain relief. There were no contraindications to percutaneous interventions or blocks such as coagulopathy, hence, diagnostic blocks of nervous structures or radiofrequency denervation were important in this patient. However, he was unable to assume the appropriate position for neural blocks.

We prescribed clonazepam 0.5 mg/day as an analgesic and anxiolytic agent. On the day after first intake of clonazepam, his pain during movement was dramatically relieved, and 3 days later, the patient was able to sit up in bed and continue cancer treatment. The adverse effects of clonazepam, such as drowsiness and dizziness, were tolerable. Fentanyl could be reduced to 1 mg with a patch. Pain improved with treatment of the MM, clonazepam was not needed 3 weeks later, and the opioid analgesics were discontinued 2 months later.

## Discussion

To the best of our knowledge, this is the first report demonstrating the efficacy of clonazepam in the management of pain caused by VCFs. VCFs are the most common complication of osteoporosis. In the United States, 700,000 patients are newly diagnosed with VCFs per year [2]. VCFs are also caused by malignant tumors and hematological malignancies. In particular, 70% of the patients with MM are affected at some stage by osteolytic diseases of the vertebrae [3]. Bone pain might impede mobility and a decline in activities of daily life. In some cases, VCFs fail to heal and may lead to the development of chronic and intractable pain. In a previous study, 46% of patients who underwent conservative treatment for acute VCFs did not receive sufficient pain relief at 12 months [4].

The management of VCFs should include pain management and activity modification. In pain management of VCFs, analgesics such as non-steroidal anti-inflammatory drugs (NSAIDs), acetaminophen, and opioids are generally used. However, MM can sometimes cause renal dysfunction. Therefore, NSAIDs are not recommended for pain management in such patients [5]. Adjuvant analgesics, including antidepressants and muscle relaxants, are also prescribed for acute low back pain due to VCFs. Systematic reviews have evidenced that NSAIDs and muscle relaxants are effective in the management of acute low back pain [6]. However, weak evidence was found for the efficacy of benzodiazepines in managing acute low back pain.

This patient had severe low back pain on movement. NSAIDs were contraindicated due to renal dysfunction caused by MM. Opioids were ineffective for his severe breakthrough pain. Thus, co-analgesics or intervention treatments were needed.

Diagnostic blocks of nervous structures or radiofrequency denervation were important in this patient. However, he was unable to assume the appropriate position for neural blocks [7].

Percutaneous vertebroplasty (PV), including balloon kyphoplasty (BKP) should be considered. BKP is a procedure used in the treatment of VCFs in which cement is injected into a fractured vertebral body [8]. BKP is more effective and safe compared to conservative treatment for pain caused by VCF due to osteoporosis and malignant diseases [9, 10]. Particularly, in patients with MM experiencing severe pain at the fracture site, PV and BKP should be indicated within the early phase of the disease [11]. However, the analgesic effect of PV and BKP in patients with facet joint pain caused by VFCs remains controversial.

Facet joint pain is one of the most common causes of chronic low back pain. The prevalence of facet joint pain in young adults with chronic low back pain is 10–15% [12] and increases in older patients. A facet joint is formed by articulation between the inferior articular process of the upper vertebra and the superior articular process of the lower vertebra. Each facet joint contains a synovial membrane, hyaline cartilage surfaces, and a fibrous capsule. The lumbar facet joint is innervated by the medial branches of the posterior ramus of the spinal nerve [13]. In addition, the medial branches innervate the multifidus and interspinous muscles. Facet joint capsular irritation may result in reflex spasm of the multifidus and other paraspinal muscles [14, 15]. Interventional procedures targeting the facet joint play a key role in the diagnosis and treatment of facet joint pain.

There are case reports of VCF causing facet joint pain [16]. The region of the patient’s pain was not the same as the dermatome of the lumbar spine root. CT and MRI revealed no findings of nerve compression and a collapse in the facet joints. His pain indicated a muscle spasm in the paravertebral region on movement, and it was consistent with the area of pain caused by facet joint pain [12]. Therefore, we considered his pain to be a facet joint pain.

As an analgesic selection, other types of adjuvant analgesics such as antidepressant or other types of opioids were considered [6]. Clonazepam, a benzodiazepine drug, is usually used as an anticonvulsant. It is indicated as an adjuvant analgesic for cancer-related neuropathic pain and burning mouth syndrome [1, 17]; however, there is no evidence that clonazepam is effective for neuropathic pain and fibromyalgia [18]. As with other benzodiazepine drugs, clonazepam acts as a muscle relaxant. As previously described, it is known that muscle relaxants are effective for acute low back pain [6]. It was considered that the patient’s severe low back pain on movement might be caused by a muscle spasm due to facet joint capsular irritation, thus clonazepam might be effective as a muscle relaxant. In addition, clonazepam is also used as an anxiolytic. This patient experienced uncontrollable fear and anxiety related to the breakthrough pain. Therefore, clonazepam might mitigate the physical and psychological distress.

In conclusion, we reported a case demonstrating the efficacy of clonazepam for patients with MM experiencing severe breakthrough low back pain due to VCFs. Pain due to VCFs is sometimes caused by facet joint irritation, which is associated with paraspinal muscle spasm. Clonazepam acts as a muscle relaxant and anxiolytic, thereby inducing pain relief in this patient.

## Data Availability

Not applicable.

## Abbreviations

VCF: Vertebral compression fracture
MM: Multiple myeloma
NRS: Numerical rating scale
CT: Computed tomography
MRI: Magnetic resonance imaging
NSAID: Non-steroidal anti-inflammatory drug
PV: Percutaneous vertebroplasty
BKP: Balloon kyphoplasty
